# Profiling Dopamine-Induced Oxidized Proteoforms of β-synuclein by Top-Down Mass Spectrometry

**DOI:** 10.3390/antiox10060893

**Published:** 2021-06-01

**Authors:** Arianna Luise, Elena De Cecco, Erika Ponzini, Martina Sollazzo, PierLuigi Mauri, Frank Sobott, Giuseppe Legname, Rita Grandori, Carlo Santambrogio

**Affiliations:** 1 Department of Biotechnology and Biosciences, University of Milano-Bicocca, 20126 Milan, Italy; a.luise1@campus.unimib.it (A.L.); rita.grandori@unimib.it (R.G.); 2 Department of Neuroscience, Scuola Internazionale Superiore di Studi Avanzati (SISSA), 34136 Trieste, Italy; edececco@sissa.it (E.D.C.); msollazz@sissa.it (M.S.); legname@sissa.it (G.L.); 3 ELETTRA-Sincrotrone Trieste S.C.p.A, Basovizza, 34149 Trieste, Italy; 4 Department of Materials Science, University of Milano-Bicocca, 20125 Milan, Italy; erika.ponzini@unimib.it; 5 Institute of Biomedical Technologies, National Research Council of Italy, Segrate, 20090 Milan, Italy; pierluigi.mauri@itb.cnr.it; 6 School of Molecular and Cellular Biology and Astbury Centre for Structural Molecular Biology, University of Leeds, Leeds LS2 9JT, UK; F.Sobott@leeds.ac.uk

**Keywords:** methionine sulfoxide, human β-synuclein, dopamine oxidation, proteoforms relative quantitation, top-down mass spectrometry

## Abstract

The formation of multiple proteoforms by post-translational modifications (PTMs) enables a single protein to acquire distinct functional roles in its biological context. Oxidation of methionine residues (Met) is a common PTM, involved in physiological (e.g., signaling) and pathological (e.g., oxidative stress) states. This PTM typically maps at multiple protein sites, generating a heterogeneous population of proteoforms with specific biophysical and biochemical properties. The identification and quantitation of the variety of oxidized proteoforms originated under a given condition is required to assess the exact molecular nature of the species responsible for the process under investigation. In this work, the binding and oxidation of human β-synuclein (BS) by dopamine (DA) has been explored. Native mass spectrometry (MS) has been employed to analyze the interaction of BS with DA. In a second step, top-down fragmentation of the intact protein from denaturing conditions has been performed to identify and quantify the distinct proteoforms generated by DA-induced oxidation. The analysis of isobaric proteoforms is approached by a combination of electron-transfer dissociation (ETD) at each extent of modification, quantitation of methionine-containing fragments and combinatorial analysis of the fragmentation products by multiple linear regression. This procedure represents a promising approach to systematic assessment of proteoforms variety and their relative abundance. The method can be adapted, in principle, to any protein containing any number of methionine residues, allowing for a full structural characterization of the protein oxidation states.

## 1. Introduction

The oxidation of proteins at methionine residues (Met) to form methionine sulfoxides (MetO) is a reversible post-translational modification (PTM) known to regulate different cellular functions, ranging from signaling to enzymatic activity and protein degradation [1,2,3,4]. The control of important biological processes is ensured by a subtle tuning of Met redox status, thanks to the action of enzymes such as monooxygenases of the MICAL family or methionine sulfoxide reductases (Msrs) [2,3,4]. Besides this involvement in physiological conditions, MetO formation is also commonly related to the excessive production of reactive oxygen species characterizing several conditions of cellular oxidative stress [5]. For this reason, MetO is frequently associated to alteration of proteostasis and development of protein aberrant states, such as amyloid aggregation and neurodegeneration [6,7,8].

Among the diseases related to Met oxidation, synucleinopathies represent very severe pathologies for human health [9]. The synuclein family consists of three intrinsically disordered proteins (IDPs): α-synuclein (AS), β-synuclein (BS) and γ-synuclein (GS) [10]. Oxidized versions of both AS and GS are known to promote protein aggregation, and are likely involved in the etiology of Parkinson’s disease (PD) and dementia with Lewy bodies (DLB) [11,12,13,14,15]. Under oxidative stress conditions, the binding of dopamine (DA) to AS causes oxidation of Met residues, with important implication on the protein structural properties and on fibril accumulation in PD and DLB [11,13,15,16,17]. Despite the high sequence similarity with AS (78%) [10], BS has been shown to have a reduced tendency to form amyloids, and has long been considered just an inhibitor of AS aggregation [18,19,20]. Recent reports, however, highlighted that in several in vivo models BS has an enhanced neurodegenerative potential in the presence of DA [21,22,23]. Moreover, some BS mutations identified in DLB patients promote its amyloid aggregation [24,25]. Interestingly, one of the aforementioned mutations (Val70Met) introduces an additional Met residue to the four already present in BS sequence (namely, Met1, Met5, Met10 and Met112). To date, a potential oxidative effect of DA on BS Met residues has not been documented yet.

Several factors contribute to make the investigation of the pathways leading to Met oxidation particularly challenging. The available anti-MetO antibodies are poorly specific [26]. In addition, oxidation events on Met residues are often linked to multiple intra- or inter-protein modifications, leading to a complex network of functionally-related PTMs. For instance, the oxidation of multiple Met residues modulates the activity of several ion channels, conferring to the cell a fine adaptation capability to different oxidative stress conditions [27,28]. Polypeptides containing MetO residues are frequently subjected to other kinds of modification events (such as phosphorylation, ubiquitination and acetylation), suggesting a functional coupling of these PTMs [29]. In particular, Met oxidation at specific residues has been proposed as a control mechanism of the phosphorylation state of several proteins [29,30], highlighting a probable crosstalk between these two modifications in many regulatory systems [31]. A correlation between the two PTMs seems to hold also for synucleinopathies, since oxidized GS and phosphorylated AS have been shown to colocalize in patients’ brains [14]. Therefore, the in-depth understanding of the regulatory mechanisms involving multiple and interconnected modifications depends on the complete description of the proteoform patterns populated by the protein.

Mass spectrometry (MS), thanks to its unique analytical power and high throughput, is one of the principal techniques adopted for PTMs characterization. To this end, a “bottom-up” approach is commonly employed, where the peptides deriving from an enzymatic digestion of the proteins are fragmented into the spectrometer, either by collision with an inert gas (collision-induced dissociation, CID; higher energy collisional dissociation, HCD) or by electron-driven methods (electron-transfer dissociation, ETD; electron-capture dissociation, ECD) [26,32,33]. The resulting fragmentation spectra (MS/MS) allow determining the nature and the position of each modification along the polypeptide chain, but fail assessing proteoforms profiles, since the information on coexisting PTMs is usually lost as a consequence of the digestion step. On the other hand, isolation and fragmentation of intact protein ions by MS (the “top-down” approach) provide mass and relative abundance of distinct protein variants, and the combined information obtained from MS and MS/MS spectra allows the identification of species with multiple PTMs [34,35,36,37]. However, profiling isobaric proteoforms remains challenging [38]. Top-down MS is also more demanding than bottom-up MS in terms of experimental procedures and data analysis, especially for “-omics” applications [32,37,39].

In this work, the oxidation of BS has been investigated by top-down MS, in order to obtain insights similar to the ones documented for AS. The effects on BS of prolonged incubation in the presence of DA have been studied analyzing the eventual formation of MetO patterns and assessing the variety of proteoforms populated. To this end, a novel approach in the processing of top-down MS data, based on the quantitation of isobaric variants from MS/MS spectra, is here proposed.

## 2. Materials and Methods

### 2.1. Chemicals and Proteins

Ammonium acetate, DA, acetonitrile and formic acid were purchased from Sigma Aldrich (St. Louis, MO, USA). The gene encoding for wild type human BS without any tag (average mass 14,287.88 g/mol) was cloned from the total cDNA of the frontal cortex of a non-neurodegenerative patient (MRC Edinburgh Brain Bank). The protein was expressed in recombinant form and purified as previously described for AS [40].

### 2.2. BS Incubation and MS Analysis

Samples containing 70 µmol/L BS were incubated for 72 h at 37 °C in 50 mmol/L ammonium acetate, pH 7.0, under mild agitation (400 rpm) in the presence of 2 mmol/L DA, as previously described for AS [17]. For BS-DA binding analysis, an aliquot of the sample was measured under native conditions within a few minutes after resuspension. For protein oxidation analysis, aliquots were taken at selected time points, diluted to 10 µmol/L protein concentration in 40% acetonitrile (*v*/*v*), 0.1% formic acid (*v*/*v*) and measured. MS experiments were performed on an Orbitrap Fusion mass spectrometer equipped with a static nano-electrospray ion source (Thermo Fisher Scientific, Waltham, MA, USA), employing metal-coated borosilicate capillaries with a medium-length emitter tip of 1-µm internal diameter (Thermo Fisher Scientific, Waltham, MA, USA). The main instrumental parameters were set as follows: resolving power at *m*/*z* 200, 120,000; IRM pressure, 3 mTorr (intact protein mode); ion spray voltage, 1.1–1.2 kV; ion-transfer tube temperature, 275 °C; in-source fragmentation, 0 V (non-denaturing conditions) or 45 V (denaturing conditions); AGC target, 4 × 10^5^; maximum injection time, 100 ms. Tandem MS analysis at selected charge states and oxidation extents (OEs) was performed by ETD fragmentation, employing a reaction time of 20 ms and an isolation width of 0.5 *m*/*z*. Final spectra were obtained by averaging the signal over 30 s acquisition.

### 2.3. Relative Quantitation of Oxidized Proteoforms

In theory, 16 oxidized proteoforms are possible for BS, depending on the oxidation state of each methionine residue. Table 1 reports a list of the proteoforms with the corresponding OE values and the nomenclature adopted in this work.

OEs fractional amounts are subjected to the constraint:

*f*(0) + *f*(1) + *f*(2) + *f*(3) + *f*(4) = 1 (1)

These fractions are obtained from the relative intensities of BS peaks in the full-scan MS analysis. In the context of intermediate OE values (1, 2 and 3), relative quantitation of the isobaric proteoforms is obtained by the analysis of the c- and z-fragments generated by ETD fragmentation. We define *R^n^_c,i_* (and *R^n^_z,i_*) as the relative intensity of the c-fragment (and z-fragment) deriving from ETD fragmentation after residue *i* and containing *n* oxidized sites. Let us consider, for instance, the c-fragment originated by fragmentation at the C-terminal side of residue 12. The relative intensity of the mono-oxidized variant for this fragment is:

*R*^1^*_c,_*_12_ = *I*^1^*_c,_*_12_/Σ*_n_*(*I^n^_c,_*_12_) (2)

For each OE value, it is possible to write a system of linear equations deriving from the methionine-containing fragments detected by tandem MS experiments. The unknowns of this system are represented by the relative amounts of the proteoforms compatible with the given OE state.

As an example, let us consider OE = 1. There are four possible proteoforms (Table 1), which are subjected to the following constraint on their relative amounts:

*r*(1,0,0,0) *+ r*(0,1,0,0) *+ r*(0,0,1,0) *+ r*(0,0,0,1) *=* 1 (3)

The system of equations for these proteoforms, expressed in matrix notation, is:

(4) $$\left(\begin{array}{cccc} 1 & 0 & 0 & 0\\ 1 & 1 & 0 & 0\\ 1 & 1 & 1 & 0\\ 0 & 1 & 1 & 1\\ 0 & 0 & 1 & 1\\ 0 & 0 & 0 & 1 \end{array}\right)\cdot \left(\begin{array}{c} r\left(1,0,0,0\right)\\ r\left(0,1,0,0\right)\\ r\left(0,0,1,0\right)\\ r\left(0,0,0,1\right) \end{array}\right)=\left(\begin{array}{c} R_{c,i}^{1}\\ R_{c,j}^{1}\\ R_{c,k}^{1}\\ R_{z,i}^{1}\\ R_{z,j}^{1}\\ R_{z,k}^{1} \end{array}\right)$$

where: 1 *≤ i ≤* 4, 5 *≤ j ≤* 9, and 10 *≤ k ≤* 111.

The system is solved combining Equations (3) and (4) and performing a multiple linear regression analysis. The fractional amounts of the proteoforms with OE = 1 is finally obtained by:

(5) $$\left(\begin{array}{c} f\left(1,0,0,0\right)\\ f\left(0,1,0,0\right)\\ f\left(0,0,1,0\right)\\ f\left(0,0,0,1\right) \end{array}\right)=f\left(1\right)\cdot \left(\begin{array}{c} r\left(1,0,0,0\right)\\ r\left(0,1,0,0\right)\\ r\left(0,0,1,0\right)\\ r\left(0,0,0,1\right) \end{array}\right)$$

The complete list of equations for all the OE values is reported in Appendix A. Data analysis was performed on three independent set of experiments, employing the software FreeStyle (Thermo Fisher Scientific, Waltham, MA, USA) to process MS and MS/MS spectra, ProSight Lite [41] to identify ETD fragments and OriginPro 2020 (OriginLab Corporation, Northampton, MO, USA) to perform multiple linear regression.

## 3. Results

### 3.1. BS Conformational Ensemble and DA-Interaction

The conformational properties of BS and its interaction with DA have been investigated by MS under non-denaturing conditions (native MS). Native MS allows for simultaneous detection of conformational components, based on charge-state distribution analysis, and non-covalent complexes, based on mass shift relative to the free forms. These features support analysis of combined folding and binding equilibria and are particularly useful for IDP studies, where conventional methods for structural characterization are heavily challenged [42,43].

**Figure 1:**
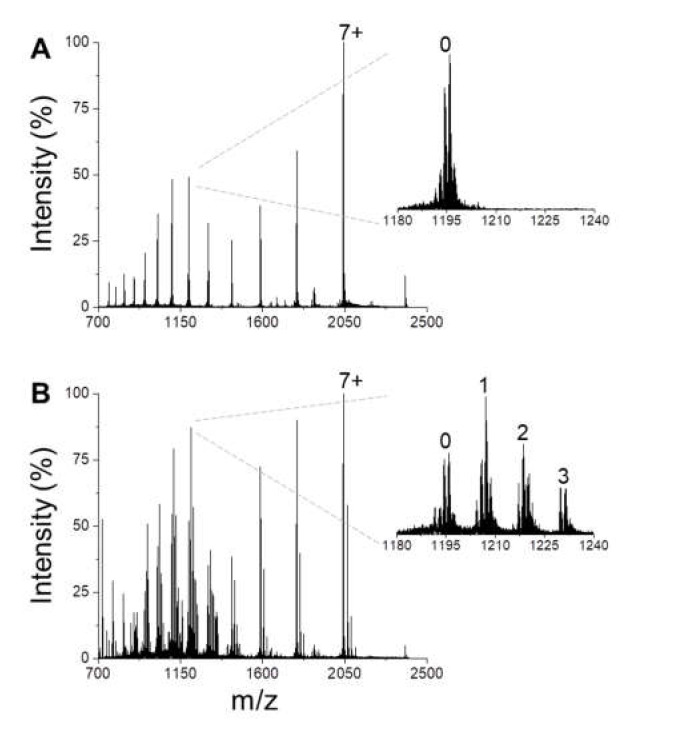
Native MS spectrum of BS in the absence (**A**) and the presence (**B**) of DA (BS:DA molar ratio 1:28). The most intense peak is labeled by the corresponding charge state. The insets show an enlargement of charge state 12+, where peaks are labeled by the number of non-covalently bound DA molecules.

**Figure 2:**
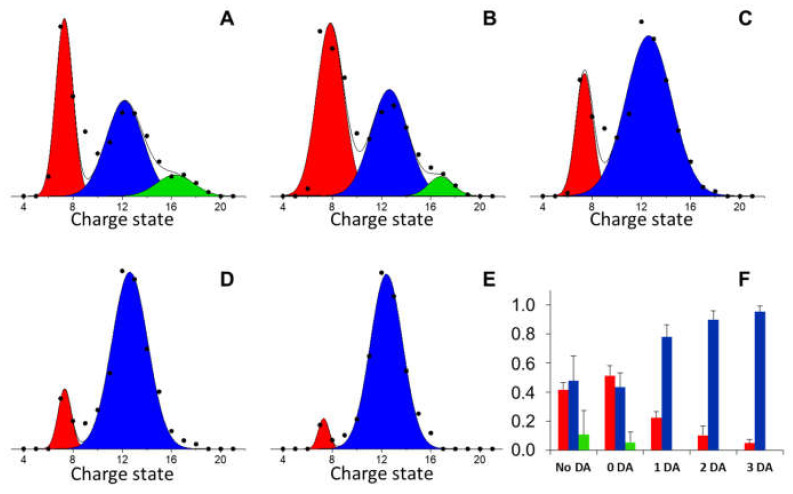
(**A**–**E**) Multiple-peak Gaussian fit of native MS spectra of Figure 1, in the absence (**A**) and in the presence (**B**–**E**) of DA, using only the signals corresponding to specific BS:DA stoichiometry values (1:0, (**B**); 1:1, (**C**); 1:2, (**D**); 1:3, (**E**)). (**F**) Fractional amount of each conformational component, assessed by the area subtended by the corresponding Gaussian curve (red, compact conformers; blue, partially extended conformers; green, extended conformers).

In the absence of ligands, the native MS spectrum of BS highlights the presence of a multimodal charge-state distribution, reflecting a heterogeneous conformational ensemble, with several components of distinct structural compactness (Figure 1A). The existence of non-globular conformers under non-denaturing conditions results from the disordered nature of BS, and not from experimentally-induced unfolding. In the presence of DA, several new peaks appear in the MS spectrum (Figure 1B), corresponding to BS-DA complexes with a protein-ligand ratio ranging from 1:1 to 1:3 (Figure 1B, inset), as already documented for AS-DA interaction [16]. The multi-Gaussian deconvolution of the spectra shows that, in the absence of DA, BS conformers populate three main conformational states (Figure 2A,F), while DA-complexes accumulate at charge states 11+ to 16+, which correspond to BS conformers of intermediate compactness (Figure 2B–F). This selectivity for the partially unfolded states has been reported also for AS-DA interaction, where it has been suggested as a possible determinant of the formation of toxic oligomers [16]. In the case of BS, however, a lower fractional amount of complexes seems to be present under similar experimental conditions [16]. This behavior is likely due to a lower DA affinity for BS relative to AS, in agreement with NMR data [23].

### 3.2. BS Oxidation Kinetics Induced by DA

Aliquots of the BS sample have been taken at several time points during the incubation in the presence of DA. These aliquots have been diluted and injected into the mass spectrometer under denaturing conditions, in order to disassemble BS-DA complexes, reduce the adducts typical of native MS electrospray (H_2_O, Na^+^, etc.) and optimize the detection of BS proteoforms. Thanks to the very high resolving power of the Orbitrap instrument employed, intact protein species are sorted at the level of isotopic distribution, allowing for a straightforward identification of charge and mass of each detected ion.

**Figure 3:**
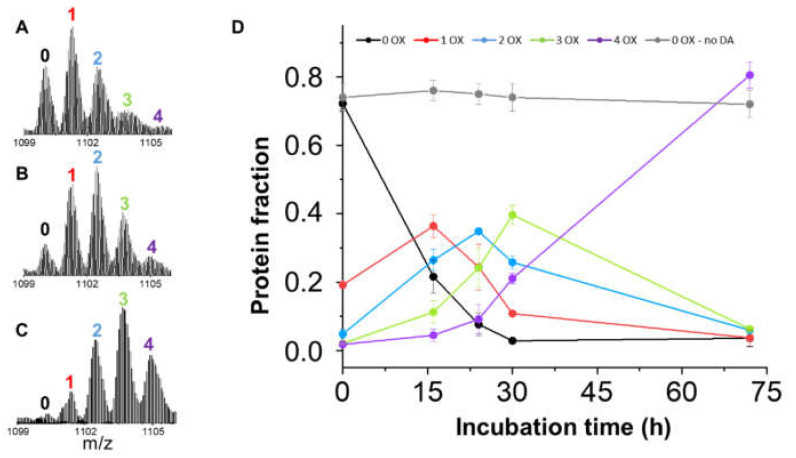
(**A**–**C**) MS spectra of BS 13+ charge state after incubation with DA for 16 h (**A**), 24 h (**B**) and 30 h (**C**). Peaks are labeled according to the OE value of the protein. (**D**) OE values as a function of the incubation time in the presence of DA (0, black; 1, red; 2, blue; 3, green; 4, magenta). The fractional amount of non-oxidized BS during the incubation in the absence of DA is shown in gray.

Distinct peaks corresponding to different PTM content are detected for each charge state. Figure 3A–C shows the details for the most intense charge state in the spectrum (13+) after variable incubation time in the presence of DA. These peaks highlight the presence of BS species with a 16 Da shift in molecular weight, indicating protein oxidation. Oxidized species do not accumulate in the absence of DA (Figure 3D), indicating that BS oxidation is induced by the presence of the ligand and is not an artefact of the electrospray process [44]. This hypothesis is supported by the evidence of H_2_O_2_ formation in the presence of DA [45], although other indirect effects, e.g., mediated by DA-induced conformational changes, cannot be ruled. The relative abundances of these species are almost constant among the charge states that populate the MS spectrum (Figure S1). Therefore, the fractional amounts of the species populating the 13+ charge state have been employed to determine BS oxidation kinetics, monitoring OE variation in time (Figure 3D). A maximum OE value of 4 has been detected under the employed experimental conditions, suggesting that oxidation takes place at the level of the 4 Met residues of the protein. Intermediate OE values (1, 2 and 3) accumulate along the incubation (15–30 h). However, at the highest time point tested (72 h) more than 80% of BS molecules present an OE value of 4. The time evolution of BS oxidized species is similar to the one shown by AS under similar experimental conditions [17], suggesting that DA promotes MetO formation by a similar mechanism for the two proteins.

### 3.3. Quantitation of BS Oxidized Proteoforms

Besides quantitation of the OE levels, profiling oxidation patterns requires full characterization of the isobaric proteoforms populating the intermediate states (OE values 1, 2, 3), which are not distinguishable by full-scan MS. All the possible configurations, differing for the positions of MetO residues, are listed in Table 1, for each OE value. ETD was used as a fragmentation method that works efficiently at high charge states and mainly breaks at the backbone level, preserving the modifications. These fragments are therefore informative of the oxidized state of specific Met residues in the context of each OE value.

**Figure 4:**
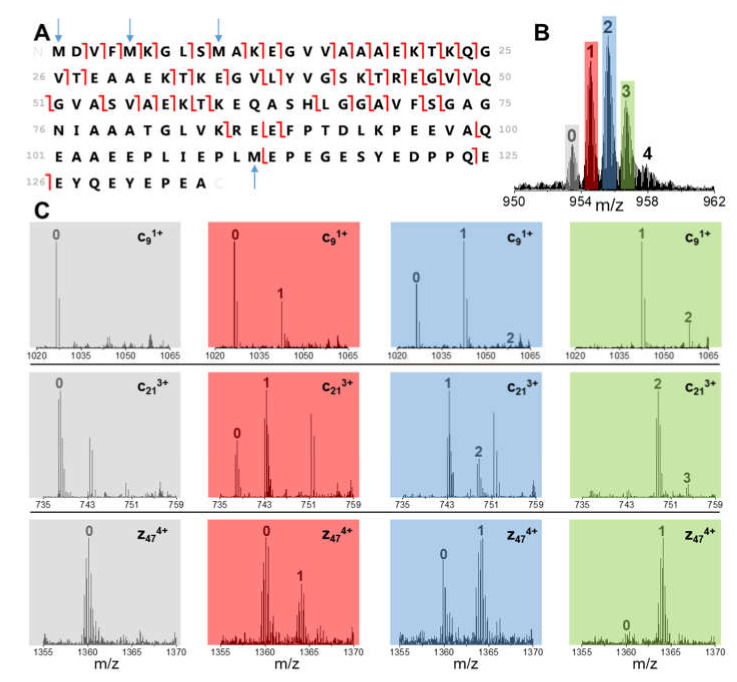
(**A**) Identified fragments from ETD analysis of the 15+ ion of non-oxidized BS. Blue arrows indicate the position of Met residues. (**B**) Precursor ion isolation of specific OE values for BS 15+ charge state after 24 h incubation. (**C**) Enlargements of MS/MS spectra acquired from (**B**) at different *m*/*z* values, in order to assess the oxidation state of selected c- and z-fragments at distinct OE values (same color code as (**B**)). Peaks are labeled by the number of oxidized residues.

Figure 4A shows the c- and z-fragments identified in the MS/MS spectrum of non-oxidized BS 15+ charge state (before incubation with DA). The latter charge state has been selected for all the ETD measurements for best fragmentation performance as a trade-off between charge state (ETD is highly dependent to the analyte net charge) and intensity.

The identified fragments containing 1, 2 or 3 Met residues have been employed to assess proteoforms distribution. The relative abundance of MetO residues in these fragments has been assessed by the fractional intensity of the corresponding oxidized peaks in MS/MS spectra for each intermediate OE value (Figure 4B,C). This information has been employed to quantify the isobaric proteoform content. For each ETD fragment, a set of linear equations describes the contribution to MetO abundances of the proteoforms compatible with the OE value under investigation. The latter equations form a system that can be solved by a multiple linear regression analysis, leading to the estimation of the fractional amount of each isobaric proteoform (see Materials and Methods section). This procedure has been adopted for the intermediate time points of BS oxidation kinetics (i.e., 16 h, 24 h and 30 h), where OE values 1, 2 and 3 accumulate in the sample.

**Figure 5:**
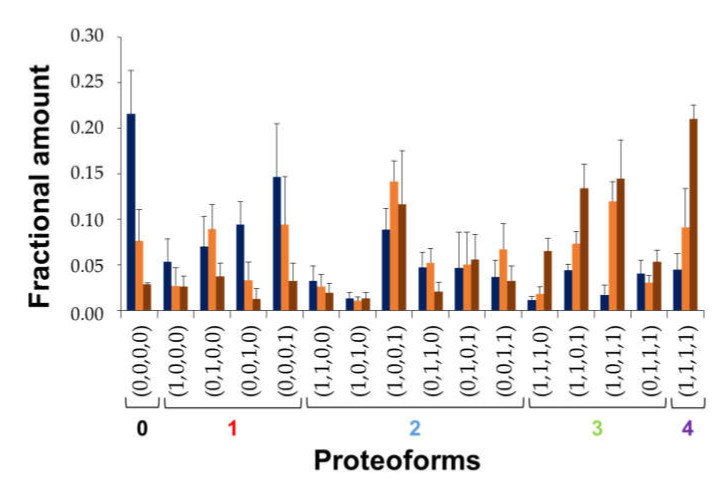
Quantitation of BS proteoforms by top-down MS after incubation in the presence of DA. Incubation time: 16 h (dark blue), 24 h (orange) and 30 h (brown). Proteoforms are grouped according to OE values. Error bars indicate the standard deviation over three independent sets of experiments.

Combining the information from MS and MS/MS spectra it was eventually possible to obtain the complete quantitation of BS proteoforms at specific incubation times with DA (Figure 5). The observed species do not highlight a dramatic trend in the evolution of their relative amounts, suggesting that BS Met residues have similar oxidative susceptibility under the conditions employed, and that no cooperative mechanisms seem to be involved in MetO formation. Nonetheless, the results show a slightly higher accumulation of the proteoforms containing oxidized Met112 at 16 h, and an increment of the proteoforms with oxidized Met112 and Met1 at longer times.

## 4. Discussion

**Figure 6:**
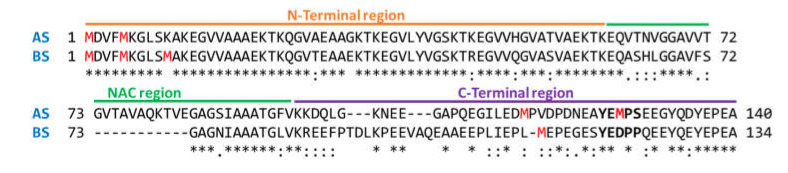
Alignment of AS and BS sequences by Clustal Omega [48]. Conserved (*), strongly similar (:) and weakly similar (.) residues are labeled. Met residues are shown in red. The AS motifs 125–129 and the BS motif 119–123 are shown in bold. The three protein regions (N-terminal, NAC and C-terminal) are also labeled.

Biomedical research in PD and DLB has mainly focused on the study of AS aggregation and the factors affecting the formation of fibrils and oligomers (mutations, PTMs, ligands, etc.). More recently, BS has been considered another important actor in the development of these diseases, even though its specific role is controversial [18,19,20,21,22,23]. This work shows that BS, similarly to AS, populates a disordered conformational ensemble and has comparable binding properties to DA, a ligand that is well known to modulate AS aggregation towards the formation of toxic oligomers [46]. These findings should be interpreted in the context of sequence homology (AS and BS are 59% identical, Figure 6), could be related to common functional properties of the two proteins in dopaminergic neurons, and are in agreement with the recent hypothesis that the two synucleins present similar neuropathological potentials despite having distinct aggregation propensities [23]. Further investigations employing other known modulators of AS aggregation (e.g., epigallocatechin-3-gallate) would help in defining BS contribution under either physiological or pathological states.

Incubation with DA promotes BS oxidation at Met residues, with a time evolution of oxidized proteoforms that does not seem to follow any particular sequential pattern. This stochastic MetO formation is likely related to the fact that the oxidative effects are dependent to DA-mediated H_2_O_2_ production in solution in the presence of oxygen [45], while not directly elicited by the interaction of the protein with the ligand. A previous paper [17] has shown that AS Met residues have comparable oxidation propensity in the presence of DA, with the only exception of Met127, belonging to the ^125^YEMPS^129^ motif. This motif, involved in DA binding [16], maps in the non-amyloid-β component (NAC) of the protein [47] and is thought to play a critical role in AS amyloid aggregation mechanism. BS lacks a Met residue in the corresponding motif (^119^YEDPP^123^, Figure 6). The interplay between sequence context, aggregation potential, MetO formation and DA binding in determining the peculiar responses of AS and BS to oxidative stress needs additional study.

The methodological approach adopted in this work allows identification and quantitation of coexisting oxidative proteoforms, including isobaric ones. The method can, in principle, be applied to any type and number of small PTMs, as well as combinations of different PTMs. In contrast to the more conventional bottom-up analysis, this approach supports the identification of the whole proteoform variety, employing fewer experimental steps and bypassing the problem of protease specificity. Differently from other previously reported top-down setups [38], distinct modification levels are analyzed individually, and the information from all the Met-containing c- and z-ions is integrated to perform proteoform quantification, allowing for all the possible combinations. On the other side, possible drawbacks of this approach might derive from the inherent limitations of intact protein handling and those related to the ETD process (i.e., unequal fragmentation propensity along the sequence and charge-state dependence of fragmentation efficiency). Nonetheless, the combination of molecule-specific and site-specific information in PTM analysis is a promising tool for in-depth proteoform profiling. The method described here will be further implemented to investigate stepwise and/or cooperative PTM-acquisition mechanisms in regulatory and signaling pathways.

## 5. Conclusions

A new approach has been described here to tackle the complexity deriving from the combination of multiple PTMs. The method is based on the principle of general validity. Nonetheless, its applicability and efficacy still need to be tested on different proteins and different PTMs, as well as by multicentric network approaches.

## Figures and Tables

**Table 1:** List of possible BS proteoforms deriving from oxidized methionine residues.

Oxidation Extent (OE)	Nomenclature	Met Residues	MetO Residues
0	(0,0,0,0)	1, 5, 10, 112	-
1	(1,0,0,0)	5, 10, 112	1
	(0,1,0,0)	1, 10, 112	5
	(0,0,1,0)	1, 5, 112	10
	(0,0,0,1)	1, 5, 10	112
2	(1,1,0,0)	10, 112	1, 5
	(1,0,1,0)	5, 112	1, 10
	(1,0,0,1)	5, 10	1, 112
	(0,1,1,0)	1, 112	5, 10
	(0,1,0,1)	1, 10	5, 112
	(0,0,1,1)	1, 5	10, 112
3	(1,1,1,0)	112	1, 5, 10
	(1,1,0,1)	10	1, 5, 112
	(1,0,1,1)	5	1, 10, 112
	(0,1,1,1)	1	5, 10, 112
4	(1,1,1,1)	-	1, 5, 10, 112

## Data Availability

The data presented in this study are available on request from the corresponding author.

## Supplementary Materials

The following are available online at https://www.mdpi.com/article/10.3390/antiox10060893/s1, Figure S1: Dependence of OE values on charge state in MS spectra.

## Appendix A

In this appendix, the analysis of isobaric proteoforms for all OE values is reported, adopting the formalism employed in the Materials and Methods section. The systems of linear equations are solved by a multiple linear regression approach; for each dataset, linear equations are generally expressed as:

$$y_{i}=\beta_{0}+\beta_{1}x_{1i}+\beta_{2}x_{2i}+\ldots +\beta_{k}x_{ki}+\varepsilon_{i}$$

where *β*_0_ is the Y intercept (in our case *β*_0_ = 0), *β*_1_, *β*_2_, …, *β*_k_ are partial coefficients and *ε_1_*, *ε*_2_, *ε_n_* are experimental variability. In the matrix notation the system is:

Y=XB+E

where:

$$Y=\left[\begin{array}{c} y_{1}\\ y_{2}\\ \vdots \\ y_{n} \end{array}\right],\;X=\left[\begin{array}{c} \;1\;\begin{array}{ccc} x_{11} & x_{12} & \ldots \end{array}\;x_{1k}\\ \;1\;\begin{array}{ccc} x_{21} & x_{22} & \ldots \end{array}\;x_{2k}\\ \vdots \;\begin{array}{ccc} \vdots \; & \;\vdots \; & \ddots \end{array}\;\vdots \\ \;1\;\begin{array}{ccc} x_{n1} & x_{n2} & \ldots \end{array}\;x_{nk} \end{array}\right],\;B=\left[\begin{array}{c} \beta_{0}\\ \beta_{1}\\ \vdots \\ \beta_{k} \end{array}\right],\;E=\left[\begin{array}{c} \varepsilon_{1}\\ \varepsilon_{2}\\ \vdots \\ \varepsilon_{n} \end{array}\right]$$

The best estimation of *B* ($\hat{B}$), obtained minimizing E, is given by:

$$\hat{B}=\left[\begin{array}{c} {\hat{\beta }}_{0}\\ {\hat{\beta }}_{1}\\ \vdots \\ {\hat{\beta }}_{k} \end{array}\right]={\left(X^{T}X\right)}^{-1}X^{T}Y$$

According to the OE value, the equations to be solved are the following:

- OE = 0

Only one proteoform is possible for this OE value, therefore:

*f*(0,0,0,0) = *f*(0)

- OE = 1

Four proteoforms are possible for this OE value, with the constrain:

*r*(1,0,0,0) + *r*(0,1,0,0) + *r*(0,0,1,0) + *r*(0,0,0,1) = 1

The system of equations for these proteoforms, expressed in matrix notation, is:

$$\left(\begin{array}{cccc} 1 & 0 & 0 & 0\\ 1 & 1 & 0 & 0\\ 1 & 1 & 1 & 0\\ 0 & 1 & 1 & 1\\ 0 & 0 & 1 & 1\\ 0 & 0 & 0 & 1 \end{array}\right)\cdot \left(\begin{array}{c} r\left(1,0,0,0\right)\\ r\left(0,1,0,0\right)\\ r\left(0,0,1,0\right)\\ r\left(0,0,0,1\right) \end{array}\right)=\left(\begin{array}{c} R_{c,i}^{1}\\ R_{c,j}^{1}\\ R_{c,k}^{1}\\ R_{z,i}^{1}\\ R_{z,j}^{1}\\ R_{z,k}^{1} \end{array}\right)$$

where: 1 *≤ i ≤* 4, 5 *≤ j ≤* 9, and 10 *≤ k ≤* 111.

Solving the system with the described method, the proteoforms fractional amounts are given by:

$$\left(\begin{array}{c} f\left(1,0,0,0\right)\\ f\left(0,1,0,0\right)\\ f\left(0,0,1,0\right)\\ f\left(0,0,0,1\right) \end{array}\right)=f\left(1\right)\cdot \left(\begin{array}{c} r\left(1,0,0,0\right)\\ r\left(0,1,0,0\right)\\ r\left(0,0,1,0\right)\\ r\left(0,0,0,1\right) \end{array}\right)$$

- OE = 2

Six proteoforms are possible for this OE value, with the constrain:

*r*(1,1,0,0) *+ r*(1,0,1,0) *+ r*(1,0,0,1) *+ r*(0,1,1,0) *+ r*(0,1,0,1) *+ r*(0,0,1,1) *=* 1

The system of equations for these proteoforms, expressed in matrix notation, is:

$$\left(\begin{array}{cccccc} 1 & 1 & 1 & 0 & 0 & 0\\ 0 & 1 & 1 & 1 & 1 & 0\\ 1 & 0 & 0 & 0 & 0 & 0\\ 0 & 0 & 1 & 0 & 1 & 1\\ 1 & 1 & 0 & 1 & 0 & 0\\ 1 & 1 & 1 & 0 & 0 & 0\\ 0 & 0 & 0 & 1 & 1 & 1\\ 0 & 1 & 1 & 1 & 1 & 0\\ 0 & 0 & 0 & 0 & 0 & 1\\ 0 & 0 & 1 & 0 & 1 & 1 \end{array}\right)\cdot \left(\begin{array}{c} r\left(1,1,0,0\right)\\ r\left(1,0,1,0\right)\\ r\left(1,0,0,1\right)\\ r\left(0,1,1,0\right)\\ r\left(0,1,0,1\right)\\ r\left(0,0,1,1\right) \end{array}\right)=\left(\begin{array}{c} R_{c,i}^{1}\\ R_{c,j}^{1}\\ R_{c,j}^{2}\\ R_{c,k}^{1}\\ R_{c,k}^{2}\\ R_{z,i}^{1}\\ R_{z,i}^{2}\\ R_{z,j}^{1}\\ R_{z,j}^{2}\\ R_{z,k}^{1} \end{array}\right)$$

where: 1 *≤ i ≤* 4, 5 *≤ j ≤* 9, and 10 *≤ k ≤* 111.

Solving the system with the described method, the proteoforms fractional amounts are given by:

$$\left(\begin{array}{c} f\left(1,1,0,0\right)\\ f\left(1,0,1,0\right)\\ f\left(1,0,0,1\right)\\ f\left(0,1,1,0\right)\\ f\left(0,1,0,1\right)\\ f\left(0,0,1,1\right) \end{array}\right)=f\left(2\right)\cdot \left(\begin{array}{c} r\left(1,1,0,0\right)\\ r\left(1,0,1,0\right)\\ r\left(1,0,0,1\right)\\ r\left(0,1,1,0\right)\\ r\left(0,1,0,1\right)\\ r\left(0,0,1,1\right) \end{array}\right)$$

- OE = 3

Four proteoforms are possible for this OE value, with the constrain:

*r*(1,1,1,0) *+ r*(1,1,0,1) *+ r*(1,0,1,1) *+ r*(0,1,1,1) *=* 1

The system of equations for these proteoforms, expressed in matrix notation, is:

$$\left(\begin{array}{cccc} 1 & 1 & 1 & 0\\ 0 & 0 & 1 & 1\\ 1 & 1 & 0 & 0\\ 0 & 1 & 1 & 1\\ 1 & 0 & 0 & 0\\ 1 & 1 & 1 & 0\\ 0 & 0 & 0 & 1\\ 1 & 1 & 0 & 0\\ 0 & 0 & 1 & 1\\ 0 & 1 & 1 & 1 \end{array}\right)\cdot \left(\begin{array}{c} r\left(1,1,1,0\right)\\ r\left(1,1,0,1\right)\\ r\left(1,0,1,1\right)\\ r\left(0,1,1,1\right) \end{array}\right)=\left(\begin{array}{c} R_{c,i}^{1}\\ R_{c,j}^{1}\\ R_{c,j}^{2}\\ R_{c,k}^{2}\\ R_{c,k}^{3}\\ R_{z,i}^{2}\\ R_{z,i}^{3}\\ R_{z,j}^{1}\\ R_{z,j}^{2}\\ R_{z,k}^{1} \end{array}\right)$$

where: 1 *≤ i ≤* 4, 5 *≤ j ≤* 9, and 10 *≤ k ≤* 111.

Solving the system with the described method, the proteoforms fractional amounts are given by:

$$\left(\begin{array}{c} f\left(1,1,1,0\right)\\ f\left(1,1,0,1\right)\\ f\left(1,0,1,1\right)\\ f\left(0,1,1,1\right) \end{array}\right)=f\left(3\right)\cdot \left(\begin{array}{c} r\left(1,1,1,0\right)\\ r\left(1,1,0,1\right)\\ r\left(1,0,1,1\right)\\ r\left(0,1,1,1\right) \end{array}\right)$$

- OE = 4

Only one proteoform is possible for this OE value, therefore:

*f*(1,1,1,1) *= f*(4)
