# Supplementary material for: Profiling Dopamine-Induced Oxidized Proteoforms of β-synuclein by Top-Down Mass Spectrometry
Source: Antioxidants (Basel). 2021 Jun 1;10(6):893. doi: 10.3390/antiox10060893 (PMC8226665; doi:10.3390/antiox10060893)
Supplement: Supplementary file 1 [file antioxidants-10-00893-s001.zip › antioxidants-1189997-supplementary.pdf]

# Profiling dopamine-induced oxidized proteoforms of β-synuclein by top-down mass spectrometry

**Arianna Luise[1], Elena De Cecco[2], Erika Ponzini[3], Martina Sollazzo[2], PierLuigi Mauri[4], Frank Sobott[5], Giuseppe Legname[2], Rita Grandori[1], and Carlo Santambrogio[1,*]**

[1] Department of Biotechnology and Biosciences, University of Milano-Bicocca, Piazza della Scienza 2, 20126 Milan, Italy; a.luise1@campus.unimib.it (A.L.), rita.grandori@unimib.it (R.G.), carlo.santambrogio@unimib.it (C.S.)

[2] Department of Neuroscience, Scuola Internazionale Superiore di Studi Avanzati (SISSA) and ELETTRA-Sincrotrone Trieste S.C.p.A, 34136 Trieste, Italy; edececco@sissa.it (E.D.C), legname@sissa.it (G.L.)

[3] Department of Materials Science, University of Milano-Bicocca, Via Roberto Cozzi 55, I-20125 Milan, Italy; erika.ponzini@unimib.it (E.P.)

[4] Institute of Biomedical Technologies, National Research Council of Italy, Segrate, 20090 Milan, Italy; pierluigi.mauri@itb.cnr.it (P.M.)

[5] Astbury Centre for Structural Molecular Biology, University of Leeds, Leeds LS2 9JT, United Kingdom; F.Sobott@leeds.ac.uk (F.S.)

* Correspondence: carlo.santambrogio@unimib.it; Tel.: +39 02 6448 3395

## SUPPLEMENTARY MATERIALS

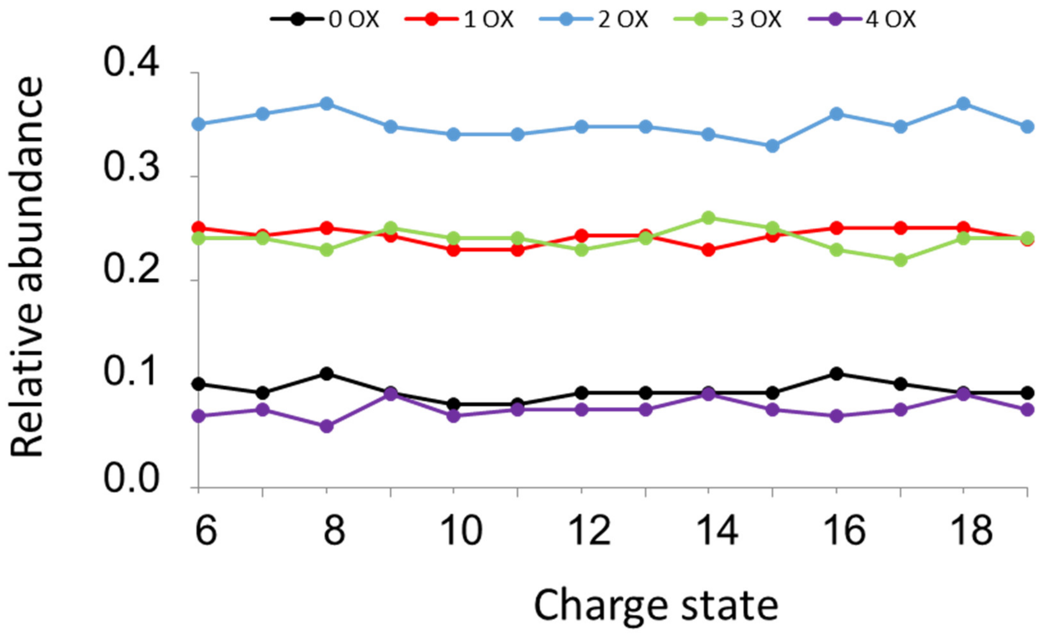


**Figure S1.** *Dependence of OE values on charge state in MS spectra*. Relative abundance of OE values *vs* charge state in MS spectra under denaturing conditions, after 24 h incubation of BS in the presence of DA.
